# An assessment of the capability of ChatGPT in solving clinical cases of ophthalmology using multiple choice and short answer questions

**DOI:** 10.1016/j.aopr.2024.01.005

**Published:** 2024-01-19

**Authors:** Anjana Christy Alexander, Suprithy Somineni Raghupathy, Krishna Mohan Surapaneni

**Affiliations:** a Department of Ophthalmology, Panimalar Medical College Hospital & Research Institute, Varadharajapuram, Poonamallee, Chennai, Tamil Nadu, India; b Department of Biochemistry, Panimalar Medical College Hospital & Research Institute, Varadharajapuram, Poonamallee, Chennai, Tamil Nadu, India; c Department of Medical Education, Panimalar Medical College Hospital & Research Institute, Varadharajapuram, Poonamallee, Chennai, Tamil Nadu, India

## Dear editor,

Artificial Intelligence (AI) has emerged as a transformative force across various domains, including healthcare and medical education.^1^ One prominent example of AI in the realm of natural language processing is ChatGPT, a chatbot developed by OpenAI.^2^ ChatGPT generates contextually relevant and coherent responses to a wide array of textual prompts, showcasing its potential in applications ranging from language understanding to problem-solving.^3^

In the context of healthcare, AI's integration has witnessed significant advancements, with applications extending to clinical decision support, diagnostics, and medical education.^4^ Ophthalmology, as a specialized field within medicine, presents unique challenges in diagnosis and management. Ophthalmic conditions often demand a keen understanding of nuanced symptoms, anatomical structures, and varied treatment approaches. Leveraging AI Chatbots like ChatGPT to address ophthalmological questions holds promise for enhancing medical education and potentially contributing to improved clinical decision-making in this intricate field.^5^ The Foreign Medical Graduate Examination (FMGE) serves as a benchmark for evaluating the competency of international medical graduates seeking to practice medicine in India.^6^ In this study, we focus on leveraging ChatGPT version 3.5 and 4.0 capabilities to answer ophthalmology multiple choice questions from the FMGE and further with more short answer questions, examining its efficacy in providing accurate responses to complex clinical cases.

## 1 Methods

ChatGPT 3.5 and 4.0 were used for this assessment. Ten clinical case scenarios which were asked in the previous year FMGE were obtained from an online platform called "*EXAMS.DMAEDU*" covering a diverse range of ophthalmological conditions, each with four multiple-choice options.^7^ Further, 10 higher-order thinking multiple choice questions and 10 short answers of clinical reasoning type were chosen from an online resource.^8^ The clinical conditions covered in the FMGE were - Conjunctivitis, Retinitis in HIV, Optic nerve pathology, Uveitis, Periorbital Cellulitis, loss of visual acuity, Retinal vein pathology, Retinal artery pathology, Papilledema and Cataract. The higher-order thinking MCQ and short answer questions converted the management, etiology and complications aspects of diverse ophthalmic conditions. The questions were presented to ChatGPT 3.5 and 4.0, and the responses were generated. Owing to the "Black-box" imaginary of artificial intelligence, to eliminate the possibility that the results of the GPT were obtained directly through an online search engine, each response was generated twice. The generated responses were then cross verified with the standard key provided. The analysis focused on evaluating the accuracy of ChatGPT version 3.5 and 4.0 responses by comparing them to the correct answers specified in the official key provided.

### 1.1 Sample questions used for this evaluation

#### 1.1.1 FMGE MCQ^7^

Nagash Natty 4-year-old child is taken by his mother to the Emergency Department as he is having pain around his right eye. He is febrile with a temperature of 38.5 ​C. This swelling started two days ago with a gradual onset. On examination, there is a tender, erythematous swelling around his right eye. **What is the SINGLE most likely diagnosis?**

• *Allergic reaction*
• *Foreign body*
• *Conjunctivitis*
• *Periorbital cellulitis*

#### 1.1.2 Higher order thinking MCQ^8^

You are on duty in the emergency center when an 18 years old high school student comes in because of pain, tearing, sensitivity to light, and blurred vision in his right eye. His symptoms began sometime that afternoon. Earlier, he had been working on his car and he remembers something flying into his right eye while he was trying to knock a rivet off the chassis with a hammer and chisel. You examine his eye and take visual acuity measurements. You determine that visual acuity is 6/18in the right eye and 6/6 in the left eye. There is some conjunctival hyperemia. The pupil of the right eye seems to be peaked and pointing to the 7 o'clock position of the There is a small, dark, slightly elevated body at the 7 o'clock position of the limbus. You cannot see fundus details of the right eye, but the left eye appears normal. **Which of the following would be the appropriate initial management for this situation?**

a Irrigation of the limbal foreign body.
b Application of a protective shield.
c Removal of the limbal foreign body with a cotton-tipped applicator.
d Removal of the limbal foreign body using forceps.
e A prescription for topical anesthetic (eg, proparacaine 0.5%) to relieve the patient's symptoms, with strict instructions that he return to see you if his blurred vision continues into the week.

#### 1.1.3 Short Answer Question ^8^

A 54-year-old man has early cataracts in both eyes. With glasses, the right eye cannot be corrected to better than 6/60, whereas with the left eye he can read the 6/12 line with best correction. The amount of cataract is exactly the same in each eye. Examination of the optic disc and macula, pupillary reaction, color vision, and retinal blood vessels proved entirely normal in each eye. However, the right eye appears to be turned slightly inward when you evaluate the corneal light reflex, and the patient has not experienced diplopia. Additional questioning reveals that the patient wore a patch over one eye as a child.

Why would information concerning his childhood ocular condition be relevant in this situation?

## 2 Results

In a comparative assessment of the performance of ChatGPT 3.5 and ChatGPT 4.0 across various medical examination formats, we observed some interesting findings. For FMGE (Foreign Medical Graduate Examination) Multiple Choice Questions, both ChatGPT 3.5 and ChatGPT 4 demonstrated strong capabilities, with ChatGPT 4.0 achieving a perfect score of 10/10. This indicates that ChatGPT 4.0 has an improved ability to handle a wide range of medical knowledge and can provide accurate responses to multiple-choice questions of varying complexity.

**Fig. 1:**
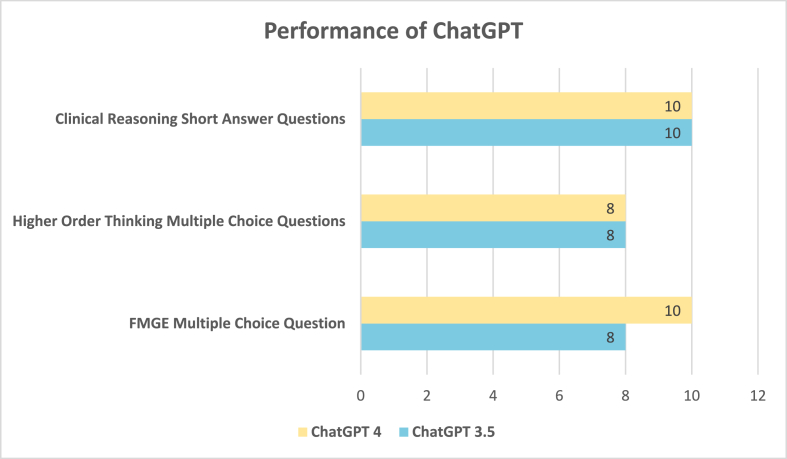
Performance of ChatGPT version 3.5 and 4.0.

In the case of Higher Order Thinking Multiple Choice Questions, both versions of the AI model, ChatGPT 3.5 and ChatGPT 4.0, displayed a commendable performance by scoring 8/10. This suggests that the improvements in ChatGPT 4.0 have positively impacted its ability to handle more complex and thought-provoking medical questions. The most notable outcome was observed in the Clinical Reasoning Short Answer Questions category. Both ChatGPT 3.5 and ChatGPT 4.0 performed exceptionally well, scoring a perfect 10/10. This implies that both versions of the AI model possess strong clinical reasoning capabilities and can provide detailed and accurate responses to complex clinical scenarios in ophthalmology. Fig. 1 shows the performance of ChatGPT 3.5 and 4.0.

However, the analysis also brought attention to noteworthy discrepancies in specific cases, such as conjunctivitis and cataracts. In the case of conjunctivitis, ChatGPT 3.5 inaccurately recommended topical antibiotics as the initial management, while the correct response advocated for the use of clean discharge using cotton wool soaked in water. Additionally, for the diagnosis of cataracts, ChatGPT 3.5 incorrectly identified the condition as macular degeneration, diverging from the officially correct response. For higher-order thinking MCQ, both versions generated erroneous responses for the management of viral conjunctivitis and injury to the eye.

## 3 Discussion

The comparative assessment of ChatGPT 3.5 and ChatGPT 4.0 reveals a dynamic landscape in the integration of AI into medical examination formats, specifically within the realm of ophthalmology. While there are notable advancements in the AI's ability to handle medical knowledge, higher-order thinking questions, and clinical reasoning, there remain areas of concern, particularly in terms of inaccuracies observed in specific cases. These findings underscore the ongoing need for vigilant development, validation, and refinement of AI models in specialized medical fields. AI should be viewed as a valuable adjunct to healthcare professionals, aiding in decision-making and knowledge dissemination, but with a recognition of its limitations. The future of AI in healthcare lies in the continuous pursuit of accuracy, reliability, and ethical practice, ultimately contributing to improved patient care and enhanced medical education within the field of ophthalmology and beyond.

In the realm of medical education, ChatGPT can serve as a dynamic knowledge resource, offering instant access to a vast repository of medical information and acting as a virtual tutor to facilitate understanding of complex medical concepts. AI-driven simulations and personalized learning paths can enhance hands-on learning and skill development, while customized assessments aid in evaluating students' knowledge. Furthermore, AI can empower healthcare professionals by assisting in diagnosing medical conditions, offering evidence-based treatment recommendations, and optimizing clinical pathways. It can reduce diagnostic errors, predict patient outcomes, and support continuing medical education, ensuring that practitioners make informed decisions and provide high-quality care.

In light of these findings, several important future directions emerge. First and foremost, there is a pressing need to address the discrepancies observed in specific cases. The inaccuracies in AI-generated responses, such as the incorrect recommendation for the initial management of conjunctivitis and the misidentification of cataracts, highlight the necessity for ongoing refinement and validation of AI models, especially in specialized medical fields like ophthalmology. Future research efforts should focus on fine-tuning AI algorithms to ensure consistently accurate and reliable responses across a wide range of clinical scenarios. Additionally, efforts should be directed towards improving AI's understanding of subtle differences in medical conditions, refining its ability to provide nuanced recommendations, and continually updating its medical knowledge base to keep pace with the ever-evolving field of medicine. Ethical considerations surrounding AI use in clinical practice, including issues of patient privacy and informed decision-making, should also be a primary focus in future development.

## 4 Conclusions

The comparative assessment of ChatGPT 3.5 and ChatGPT 4.0 within ophthalmology highlights advancements in their ability to handle medical knowledge, higher-order thinking questions, and clinical reasoning. However, concerns arise from observed inaccuracies in specific cases. This underscores the ongoing need for AI model development and validation in specialized medical fields. AI serves as a valuable tool for healthcare professionals, aiding decision-making and knowledge dissemination, but its limitations must be acknowledged. The future of AI in healthcare hinges on continuous improvement, ethical practice, and its role in enhancing patient care and medical education in ophthalmology and other specialities.

## Funding

This research did not receive any specific grant from funding agencies in the public, commercial, or not-for-profit sectors.

## Declaration of competing interest

The authors declare that they have no known competing financial interests or personal relationships that could have appeared to influence the work reported in this paper.
