# Supplementary material for: An assessment of the capability of ChatGPT in solving clinical cases of ophthalmology using multiple choice and short answer questions
Source: Adv Ophthalmol Pract Res. 2024 Jan 19;4(2):95–7. doi: 10.1016/j.aopr.2024.01.005 (PMC11043809; doi:10.1016/j.aopr.2024.01.005)
Supplement: Multimedia component 1 [file mmc1.docx]

**An Assessment to Appraise the Capability of ChatGPT in Solving Clinical Cases of Ophthalmology in Foreign Medical Graduate Examination (FMGE)**

Dear Editor

Artificial Intelligence (AI) has emerged as a transformative force across various domains, including healthcare and medical education [1]. One prominent example of AI in the realm of natural language processing is ChatGPT, a chatbot developed by OpenAI [2]. ChatGPT utilizes the GPT-3.5 architecture to generate contextually relevant and coherent responses to a wide array of textual prompts, showcasing its potential in applications ranging from language understanding to problem-solving [3].

In the context of healthcare, AI's integration has witnessed significant advancements, with applications extending to clinical decision support, diagnostics, and medical education [4]. Ophthalmology, as a specialized field within medicine, presents unique challenges in diagnosis and management. Ophthalmic conditions often demand a keen understanding of nuanced symptoms, anatomical structures, and varied treatment approaches. Leveraging AI ChatGPTs like ChatGPT to address ophthalmological questions holds promise for enhancing medical education and potentially contributing to improved clinical decision-making in this intricate field [5]. The Foreign Medical Graduate Examination (FMGE) serves as a benchmark for evaluating the competency of international medical graduates seeking to practice medicine in India [6]. In this study, we focus on leveraging ChatGPT's capabilities to answer ophthalmology questions from the FMGE, examining its efficacy in providing accurate responses to complex clinical cases.

ChatGPT 3.5 was used for this assessment. Ten clinical case scenarios which were asked in the previous year FMGE were obtained from an online platform called “*EXAMS.DMAEDU*” covering a diverse range of ophthalmological conditions, each with four multiple-choice options [7]. The clinical conditions covered were - Conjunctivitis, Retinitis in HIV, Optic nerve pathology, Uveitis, Periorbital Cellulitis, loss of visual acuity, Retinal vein pathology, Retinal artery pathology, Papilledema and Cataract The questions were presented to ChatGPT, and the generated responses were then cross-verified. The analysis focused on evaluating the accuracy of ChatGPT's responses by comparing them to the correct answers specified in the official key provided in the website.

The evaluation of ChatGPT's performance in responding to ten clinical case-based questions from the Foreign Medical Graduate Examination (FMGE) yielded an overall accuracy rate of 80%. Among the questions presented, ChatGPT demonstrated proficiency by providing correct responses for eight cases. This indicates ChatGPT's capability to comprehend and generate relevant answers in the context of ophthalmology.

However, the analysis also brought attention to noteworthy discrepancies in specific cases, such as conjunctivitis and cataract. In the case of conjunctivitis, ChatGPT inaccurately recommended topical antibiotics as the initial management, while the correct response advocated for the use of clean discharge using cotton wool soaked in water. Additionally, for the diagnosis of cataract, ChatGPT incorrectly identified the condition as macular degeneration, diverging from the officially correct response.

The study's outcomes underscore the dual nature of ChatGPT's potential in medical education, particularly in addressing ophthalmology questions from the FMGE. While the overall accuracy of 80% is promising, the identified discrepancies in responses for cases like conjunctivitis and cataract highlight the nuanced challenges AI models face in accurately interpreting specific clinical scenarios. These discrepancies emphasize the importance of cautious integration and ongoing validation of AI models in medical education. The study suggests that while AI can serve as a valuable educational tool, it should be used in conjunction with, rather than as a replacement for, clinical expertise. Future educational strategies incorporating AI should be mindful of these implications, focusing on refining models to ensure alignment with the complex realities of medical practice.

Looking ahead, the future direction for research in AI-driven medical education involves refining models like ChatGPT to address discipline-specific challenges, with a particular focus on ophthalmology. Collaborative efforts between AI developers, medical educators, and clinicians are essential to enhance the model's understanding of intricate clinical nuances. Integration of AI-driven interactive learning platforms into medical curricula, providing simulated clinical scenarios and immediate feedback, presents an exciting avenue for advancing medical education. Longitudinal studies assessing the real-world impact of AI on diagnostic accuracy and decision-making skills will be crucial, guiding continuous adaptation and improvement based on clinical feedback.

**Conflict of interest:** None to be declared

**Funding:** Nothing to Declare

**Acknowledgements:** Authors would like to extend their gratitude to the OpenAI, an American

artificial intelligence research laboratory for providing free access to ChatGPT 3.5.

**References:**

1. Chen, M., & Decary, M. (2020). Artificial intelligence in healthcare: An essential guide for health leaders. Healthcare management forum, 33(1), 10–18. <https://doi.org/10.1177/0840470419873123>
2. OpenAI. Introducing ChatGPT. Available from: <https://openai.com/blog/chatgpt>. Last accessed on December 2, 2023.
3. Briganti G. (2023). How ChatGPT works: a mini review. European archives of oto-rhino-laryngology: official journal of the European Federation of Oto-Rhino-Laryngological Societies (EUFOS) : affiliated with the German Society for Oto-Rhino-Laryngology - Head and Neck Surgery, 10.1007/s00405-023-08337-7. Advance online publication. <https://doi.org/10.1007/s00405-023-08337-7>
4. Dave, T., Athaluri, S. A., & Singh, S. (2023). ChatGPT in medicine: an overview of its applications, advantages, limitations, future prospects, and ethical considerations. Frontiers in artificial intelligence, 6, 1169595. <https://doi.org/10.3389/frai.2023.1169595>
5. Ting, D. S. J., Tan, T. F., & Ting, D. S. W. (2023). ChatGPT in ophthalmology: the dawn of a new era?. Eye (London, England), 10.1038/s41433-023-02619-4. Advance online publication. <https://doi.org/10.1038/s41433-023-02619-4>
6. Anjali, S., Sanjay, Z., & Bipin, B. (2016). India's foreign medical graduates: an opportunity to correct India's physician shortage. Education for health (Abingdon, England), 29(1), 42–46. <https://doi.org/10.4103/1357-6283.178932>
7. EXAMS.DMAEDU. Ophthalmology FMGE Case Based Questions. 2020. Available from: Last accessed on December 2, 2023.

**Supplementary File: Link to ChatGPT Response**

1. <https://chat.openai.com/share/7aa0db74-02d6-46f1-bc8f-0ff1aed44ba4>
2. <https://chat.openai.com/share/d01b75fd-b83c-4e6d-8bcb-3acb2e9c5507>
3. <https://chat.openai.com/share/f2854de1-85c9-41df-86bd-2320aae8efa3>
4. <https://chat.openai.com/share/0582c8f6-b78f-42f1-878f-57e3ca7a01ef>
5. <https://chat.openai.com/share/09c71e74-cf21-46b9-b1eb-92f797d5e643>
6. <https://chat.openai.com/share/6ddc0dd3-84ca-402e-8a8f-29cf162abe0b>
7. <https://chat.openai.com/share/f70c7706-a7b0-4ba5-a033-89e58e0d67f4>
8. <https://chat.openai.com/share/71f0e9db-13f6-4a70-b373-6ea5a3565e19>
9. <https://chat.openai.com/share/59fc0c04-b5f7-45ab-8d77-ced9c15d8e86>
10. <https://chat.openai.com/share/76f86b3f-3401-456b-a5c8-97bd389edf51>
